# External Validation of the Simple Clinical Score and the HOTEL Score, Two Scores for Predicting Short-Term Mortality after Admission to an Acute Medical Unit

**DOI:** 10.1371/journal.pone.0105695

**Published:** 2014-08-21

**Authors:** Mia Stræde, Mikkel Brabrand

**Affiliations:** 1 Department of Anaesthesiology, Sydvestjysk Sygehus Esbjerg, Esbjerg, Denmark; 2 Department of Medicine, Sydvestjysk Sygehus Esbjerg, Esbjerg, Denmark; 3 Department of Emergency Medicine, Odense University Hospital, Odense, Denmark; National Taiwan University, Taiwan

## Abstract

**Background:**

Clinical scores can be of aid to predict early mortality after admission to a medical admission unit. A developed scoring system needs to be externally validated to minimise the risk of the discriminatory power and calibration to be falsely elevated. We performed the present study with the objective of validating the Simple Clinical Score (SCS) and the HOTEL score, two existing risk stratification systems that predict mortality for medical patients based solely on clinical information, but not only vital signs.

**Methods:**

Pre-planned prospective observational cohort study.

**Setting:**

Danish 460-bed regional teaching hospital.

**Findings:**

We included 3046 consecutive patients from 2 October 2008 until 19 February 2009. 26 (0.9%) died within one calendar day and 196 (6.4%) died within 30 days. We calculated SCS for 1080 patients. We found an AUROC of 0.960 (95% confidence interval [CI], 0.932 to 0.988) for 24-hours mortality and 0.826 (95% CI, 0.774–0.879) for 30-day mortality, and goodness-of-fit test, χ^2^ = 2.68 (10 degrees of freedom), P = 0.998 and χ^2^ = 4.00, P = 0.947, respectively. We included 1470 patients when calculating the HOTEL score. Discriminatory power (AUROC) was 0.931 (95% CI, 0.901–0.962) for 24-hours mortality and goodness-of-fit test, χ^2^ = 5.56 (10 degrees of freedom), P = 0.234.

**Conclusion:**

We find that both the SCS and HOTEL scores showed an excellent to outstanding ability in identifying patients at high risk of dying with good or acceptable precision.

## Introduction

It can be a difficult task to determine the prognosis of acutely ill medical patients, and clinical scoring systems can be of assistance. For a score to be useful it needs to be easy to calculate and accurate in it's prediction. External validation (i.e. at another location than where the system was developed) in a separate cohort is a good measure of the reliability of a scoring system, and a scoring system should only be used if it has been thoroughly validated [1].

There exists quite a few different scoring systems. Some use only vital signs, others only biochemical data and some use information on prior medical illnesses [1]. The Simple Clinical Score (SCS) is based on 16 independent predictors of 30-day mortality. It includes age, blood pressure, heart rate, temperature, oxygen saturation, respiratory rate, abnormal ECG, breathless on presentation, diabetes, coma without intoxication, altered mental status, new stroke on presentation, inability to stand unaided and whether the patient was bedridden prior to the current illness, see table 1 [2]. The HOTEL score is based on Hypotension, Oxygen saturation, low Temperature, ECG changes, and Loss of independence. It predicts early mortality between 15 minutes and 24 hours from the time of admission, see table 2 [3].

**Table 1:** The Simple Clinical Score.

*Variable*	Points
*Age (years)*	
<50 for men or <55 for women	0
⩾ 50 for men and ⩾ 55 for women, but ⩽ 75 for either	2
>75 for both men and women	4
*Systolic blood pressure (mmHg)*	
>100	0
>80 and <100	2
>70 and <80	3
<70	4
Pulse rate > systolic blood pressure	2
Temperature <35°C or ⩾ 39°C	2
*Respiratory rate (per min)*	
<20	0
>20 and <30	1
>30	2
*Oxygen saturation*	
⩾ 95%	0
⩾ 90% and <95%	1
<90%	2
Breathless on presentation	1
Abnormal ECG	2
Diabetes (Type I or II)	1
Coma without intoxication or overdose	4
Altered mental status without coma, intoxication or overdose, and aged >50 years	3
New stroke on presentation	3
Unable to stand unaided, *or* a nursing home resident	2
Prior to current illness, spent some part of daytime in bed	2

**Table 2:** The HOTEL score.

*Variable*	Points
Systolic blood pressure (mmHg) <100	1
Oxygen saturation (%) <90	1
Temperature <35°C	1
Abnormal ECG	1
Unable to stand unaided	1

SCS and HOTEL were chosen, as they are the only two scoring systems that require ECG changes and loss of independence. SCS has previously been externally validated [4] [5] [6], but HOTEL has to the best of our knowledge never been externally validated. Our aim was to externally and independently validate and compare SCS and HOTEL in a cohort of acutely admitted medical patients.

## Methods

### Setting

The study was performed at Sydvestjysk Sygehus, a 460-bed regional teaching hospital. All subspecialties of internal medicine are represented. It serves a population of 220,000. The medical admission unit (MAU) has 24 beds, and 10.950 admissions per year. Two attending physicians, one in internal medicine and one in cardiology, one senior resident, and two interns staff the MAU. A physician refers all patients to the unit.

### Design and data

We conducted a prospective observational cohort study of all patients admitted through (MAU) at our hospital. All consecutive adult patients (age ≥15 years) admitted from 2 October 2008 until 19 February 2009 were included.

A nurse registered vital signs, loss of independence and demographic information. The first physician to see the patient performed ECG interpretation. In case of missing data, we tried to extract this from an electronic copy of the nurse's notes or the chart. Validation against the central hospital database was performed to minimise the risk of incomplete inclusion. A patient was excluded if one or more of the variables required for a given risk assessment tool were missing.

We defined the primary outcome as in the original articles, i.e. 1-day and 30-day mortality [3] [2]. After completed inclusion of the patients, and all the patients were either discharged or dead, mortality data were extracted from the hospital computer systems.

We performed Pearson correlation test to assess the correlation between the two scoring systems.

The data were anonymized and de-identified prior to analysis, thus informed consent was not needed. The study was approved by the Danish Data Protection Agency. Approval from an Ethics Committee was not required according to Danish law. The study is reported in accordance with the STROBE statement [7].

### Statistics

The sample size was dictated by another part of the study. In brief, the sample size was calibrated to develop and validate a risk stratification system to predict seven-day all-cause mortality (unpublished). However, this study was pre-planned.

We calculated the area under the receiver-operating characteristic curve (AUROC) to assess the discriminatory power (i.e., the ability to identify patients at highest risk of dying). An AUROC above 0.8 is said to represent excellent discriminatory power [8]. We applied the Hosmer–Lemeshow goodness-of-fit test according to Seymour et al [9] in order to asses the calibration (precision). A P-value above 0.05 indicates acceptable calibration.

Data are reported as median (inter-quartile range) or proportions whenever appropriate. Differences between patients with and without missing data were tested using the χ^2^ test or Wilcoxon rank sum test. Stata version 13.1 (StataCorp, College Station, TX, USA) was used for analyses.

## Results

A total of 3046 patients were included in the study. Among these, 26 (0.9%) died within one day and 196 (6.4%) died within 30 days. Table 3 compares Kellett's cohort from the original article [2] to our cohort.

**Table 3:** Comparison between original derivation cohort and the danish validation cohort.

	Kellett Derivation cohort (n = 6736)	Danish validation cohort (n = 3046)
Age	61.9±20.3	62.4+/−19.2
Systolic blood pressure (mmHg)	136±27	134.0+/−24.8
Diastolic blood pressure (mmHg)	76±16	78.3+/−15.0
Pulse rate (bpm)	86±20	88.2+/−22.2
Temperature (celcius)	36.4±0.8	37.0+/−0.9
Oxygen saturatoin (%)	95.4±3.9	94.8+/−4.7
Respiratory rate (per min)	20±4	19.3+/−6.2
Death within 24 hours of admission	40 (0.6%)	26 (0.9%)
Death within 30 days of admission	316 (4.7%)	196 (6.4%)
Male sex	3534 (52.5%)	1586 (52.1%)
Self-referred	1931 (28.7%)	0 (0%)
Nursing home resident	361 (5.4%)	136 (4.6%)
Unable to stand unaided, and not a nursing home resident	713 (10.6%)	573 (23.0%)
Diabetes	1066 (15.8%)	432 (14.2%)
Abnormal ECG	3933 (58.4%)	1025 (55.0%)

### Validation of SCS

**Figure 1:**
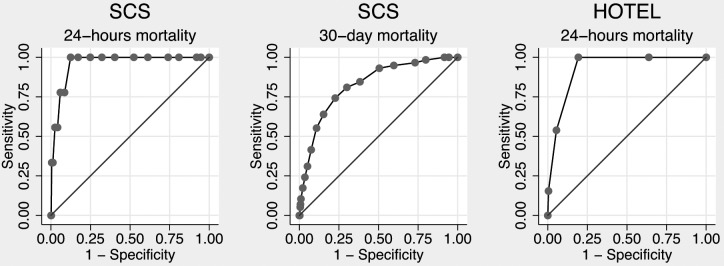
Comparison of area under the receiver operating characteristic curve of each scoring system.

**Figure 2:**
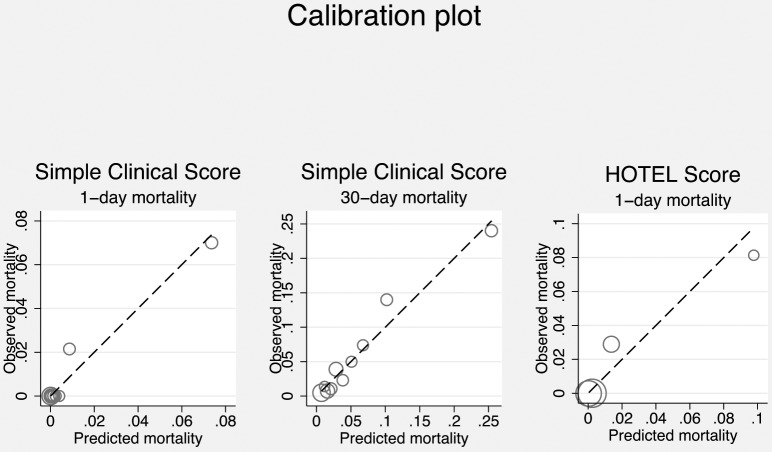
Pearson Correlation test plot.

We could calculate SCS for 1080 patients (35.5% of the cohort). Using the original formula, we found an AUROC of 0.960 (95% confidence interval [CI], 0.932 to 0.988) for 24-hours mortality and 0.826 (95% CI, 0.774–0.879) for 30-day mortality (figure 1), and goodness-of-fit test, χ^2^ = 2.68 (10 degrees of freedom), P = 0.998 and χ^2^ = 4.00, P = 0.947, respectively (figure 2). Thus, SCS had excellent to outstanding ability to identify patients at high risk of dying at both 1-day and 30-days with good precision.

### Validation of HOTEL

We could include 1470 (48.3%) patients when calculating HOTEL. Discriminatory power (AUROC) was 0.931 (95% CI, 0.901–0.962) for 24-hours mortality (figure 1) and goodness-of-fit test, χ^2^ = 5.56 (10 degrees of freedom), P = 0.234 (figure 2). Thus, HOTEL showed outstanding ability to identify patients at high risk of dying with acceptable precision.

### Correlation between SCS and HOTEL

**Figure 3:**
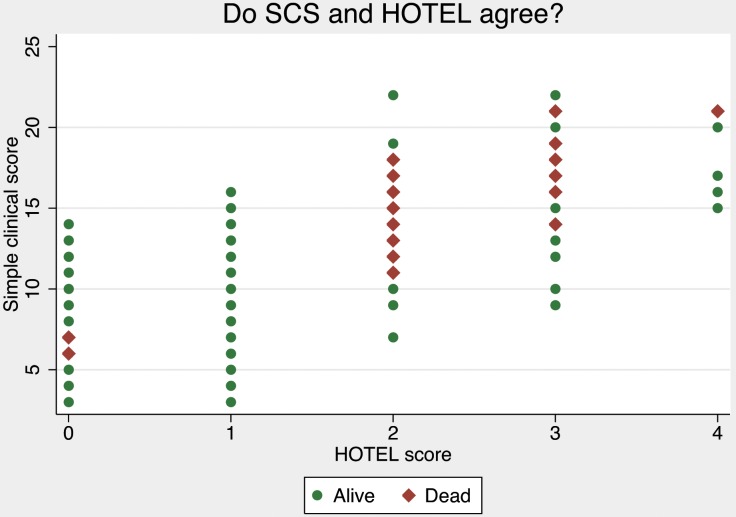
Scatter plot of SCS vs. HOTEL scores.

We found good but not perfect correlation between the two score with the Pearson test, r^2^ = 0.78 p<0.001. We also performed a scatter plot of the two scores, (figure 3).

### Missing data

1966 patients were excluded from SCS due to incomplete registration and 1576 from HOTEL; the major reasons being missing ECG (1183 vs. 1183), respiratory rate (916), loss of independence (550 vs. 550) and daytime in bed (990).

### Selection bias

We examined for significant differences between the included and excluded patients in age, gender, respiratory rate, ECG, loss of independence, daytime in bed, 24-hours mortality, 30-days mortality and Charlson comorbidity score (table 4). The Charlson score is a weighted index of comorbid disease to predict risk of 1-year mortality. It is very well validated [10], [11]. The only significant difference was found in age. For both scores, the included patients were significantly younger.

**Table 4:** Sources of selection bias in excluded patients.

	SCS				HOTEL	
	Included	Excluded	p-value	Included	Excluded	p-value
Age	66.04	60.45	p<0.0001	65.63	59.45	p<0.0001
Sex -female	519/1080	941/1966	0.919	699/1470	761/1576	0.685
24-h mortality	9/1080	17/1966	0.928	13/1470	13/1576	0.858
30-day mortality	58/1080	138/1966	0.08	-	-	-
Charlson score			0.393			0.716

The only significant difference between included and excluded patients was age. For both scores, the included patients were significantly younger. We examined for age, female sex, respiratory rate, ECG, loss of independence, daytime in bed, 24-hours mortality, 30-days mortality and Charlson comorbidity score, and found no other difference; this is demonstrated in table 4. The Charlson score is a weighted index of comorbid disease to predict risk of 1-year mortality. It is very well validated [10], [11].

## Discussion

We performed an external and independent validation of the SCS and HOTEL scores on 3046 patients. Even though we excluded quite a few patients due to incomplete data collection, we found that SCS and HOTEL both have excellent to outstanding ability to identify patients at high risk of dying with good precision.

The precision for predicting mortality is best for both scores at 24-hours (figure 1). In general, most fatalities are preceded by abnormalities in vital signs, which would raise both scores. To the experienced doctor, these predictions can be made directly from clinical observations, and the extra piece of information that the scores provide, might not change the patient's treatments. This raises the question of clinical relevancy of a score to predict 24-hours mortality. A calculation of 30-day mortality seems more relevant in daily clinical practice, since it is difficult to predict.

To compare the two scores we performed a scatter plot (figure 3). The scatter plot shows the discrepancy between the scores, which is rather large. A SCS 15 could mean a HOTEL of 1, 2, 3 or 4, and a HOTEL of 2 ranges from SCS 7 to 22. Since HOTEL is much more simple than SCS, there will of course be some differences between the two, but with a calculated correlation of r^2^ = 0.78 (p<0.001), they are not far apart. This makes both scores quite imprecise for the individual patient, and the scores seem of little value on an individual level. This reminds us that scores are developed on groups of patients, and we have to be cautious in applying them to individuals [1]. [12]

Our study has limitations. First of all, we could only calculate SCS and HOTEL in 35% and 51% of the patients respectively. This was unintended, as we designed the study specifically to validate the SCS and HOTEL scores and the staff were trained prior to the inclusion period. SCS uses 16 parameters, some of which may be difficult for the working staff to register. HOTEL is easier to use and yet still applicable to the majority of acutely ill medical patients. Secondly, we asked a physician to perform the ECG analysis instead of using an automated method as in the original article. We believe that having a physician perform the analysis reduces the risk of incorrect distinction between normal and pathological ECGs.

Many patients did not have an ECG performed. ECG is not routinely done on all patients in our unit, but only performed when there is suspicion of heart disease. This resulted in incomplete data collection from many patients and therefore exclusion. To determine the possible bias this might have added to our results, we calculated mortality for general medical patients and patients admitted to the cardiology services. The general medical patients had a 30-day mortality of 7.9% and the same was 3.9% for the cardiology patients. We found no significant differences in length of stay (LOS).

SCS has been validated in various settings, but only a few independent studies have been done [4], [5], [6]. These all point in the direction that SCS is a valid tool to use in the MAU. In a validation study of SCS on 1072 septic patients, Ghanem-Zoubi et al. found that SCS had acceptable accuracy despite the selected case-mix [6]. It has been demonstrated that SCS can be used in MAUs to predict LOS, and as a guide in finding patients at risk for intensive critical care. [4]

## Conclusions

In conclusion, both SCS and HOTEL predict mortality with acceptable precision and excellent discrimination. HOTEL seems easier to use, and with an AUROC of 0.960 in an external validation study, it seems valid for predicting early mortality in the acutely ill medical patient. The SCS is also very accurate, but more difficult to use in daily practice. Moreover, SCS predicts 30-day mortality that can be difficult to predict clinically.
